# Research on Multi-Feature Fusion and Lightweight Recognition for Radar Compound Jamming

**DOI:** 10.3390/s26041296

**Published:** 2026-02-17

**Authors:** Weiyu Zha, Jianyin Cao, Hao Wang, Wenming Yu

**Affiliations:** 1 School of Electronic and Optical Engineering, Nanjing University of Science and Technology, Nanjing 210094, China; zhawy218@njust.edu.cn (W.Z.); jianyin.cao@njust.edu.cn (J.C.); 2 Nanhu Laboratory, Jiaxing 314051, China; 3 School of Information Science and Engineering, Southeast University, Nanjing 211189, China; wmyu@seu.edu.cn

**Keywords:** radar compound jamming, lightweight recognition, low JNR, multi-feature fusion

## Abstract

To recognize radar compound jamming under complex electromagnetic environments, this paper proposes a lightweight multi-feature fusion network for compound jamming recognition. Three complementary time–frequency representations are employed to extract various features of compound jamming, which are processed by a multi-branch architecture for parallel, multi-scale feature learning. Attention mechanisms are incorporated to enhance the discriminative characteristics of jamming, and a weighted fusion strategy is adopted to integrate multi-channel features effectively. Furthermore, an improved lightweight module, GSENet, is introduced to construct the recognition network with low complexity. Experiments on simulated radar jamming datasets demonstrate that the proposed network achieves over 87% recognition accuracy for seven compound jamming types under low jamming-to-noise ratio (JNR) conditions while maintaining a parameter count below 0.14 M. These results indicate that the proposed network provides an effective trade-off between recognition performance and model complexity, making it suitable for electronic counter-countermeasure (ECCM) applications.

## 1. Introduction

The rapid advancement of electronic and communication technologies has led to an increasingly complex and congested electromagnetic environment. Within this context, and alongside the growing low-altitude economy, challenges in accurate target recognition and control have become critical issues, demanding urgent attention. As a pivotal military asset, radar systems are essential for detecting, identifying, and tracking targets under such intricate conditions [1,2,3]. Concurrently, the proliferation of modern digital radio frequency memory (DRFM) technology has intensified the competition between radar jamming and anti-jamming capabilities. In addition to active jamming, radar and sensing systems are increasingly affected by unintentional radio frequency interference (RFI) and ambient Gaussian noise, which can severely degrade parameter estimation and situational awareness [4]. Consequently, the precise recognition of jamming signals forms the foundation for effective countermeasures, highlighting the significant theoretical and practical importance of developing accurate and robust jamming recognition methodologies.

Research on radar jamming recognition has seen considerable progress. Traditional techniques are primarily categorized into likelihood-ratio-based and feature-extraction-based methods [5]. For instance, Greco et al. [6] modeled jamming signals within a cone surrounding the true target and employed a generalized likelihood ratio test (GLRT) for discrimination. However, such methods often face practical limitations due to their dependence on sufficient prior knowledge. Alternatively, feature-extraction methods first analyze and extract distinctive features of jamming signals in the time [7] or frequency domain [8], followed by classification. For example, Su et al. [9] extracted low-complexity, noise-robust features and designed a stepwise threshold process for several typical active jamming types, achieving over 98% recognition accuracy under favorable signal conditions. For compound jamming, where nonlinear coupling renders single-domain analysis ineffective, joint time–frequency analysis techniques, such as the short-time Fourier transform (STFT) [10,11], the Wigner–Ville distribution (WVD) [12,13], and the wavelet transform (WT) [14,15], are commonly adopted to extract hybrid features.

The evolution of machine learning has introduced a diverse array of classifiers into this domain. Researchers have utilized fuzzy C-means clustering for decision trees [16], support vector machines (SVMs) with polarization features [17], and variational-mode decomposition (VMD) combined with SVM for compound deceptive jamming [18]. Bayesian classifiers have also been applied for feature fusion, demonstrating high recognition rates even under low jamming-to-noise ratios (JNRs) [19]. Nevertheless, these approaches often involve computationally intensive, handcrafted feature-extraction processes, in which performance is critically contingent upon the quality and choice of features.

Significant developments in deep learning, driven by increased computational power and large-scale data availability, have revolutionized automated feature extraction and recognition [20]. In particular, convolutional neural networks (CNNs) have demonstrated exceptional prowess in automatically learning discriminative features from raw or transformed data [21,22]. Researchers have incorporated these developments into the domain of radar jamming signal processing [23,24]. Ruo-Ran [25] leveraged the exceptional nonlinear mapping capability of the BP network to extract signal features from multiple dimensions for recognition, with the classifier’s performance gradually improving as the JNR increases. Liu et al. [26] utilized STFT to conduct time–frequency analysis on nine types of jamming signals and input the resulting time–frequency images into a CNN for recognition. In [27], time-domain features were extracted using one-dimensional CNNs, while time–frequency-domain features were extracted using two-dimensional CNNs. A fusion strategy was adopted to recognize twelve variants of jamming signals through feature fusion. To improve the recognition performance of compound jamming signals, Qu et al. [28] proposed JRNet, based on robust power spectral features, which demonstrates superior recognition performance under low JNR conditions. Kong et al. [29] proposed a neural network that combines time–frequency and range-Doppler (RD) maps derived from radar data, facilitating the identification of multiple active deception jamming signals within expanded target environments and demonstrating improved accuracy and robustness compared to traditional machine learning techniques. For the reliable recognition of both classical and compound jamming signals, Zhou et al. [30] devised two deep learning models utilizing FRFT and time–frequency images. The experimental outcomes revealed an average recognition accuracy exceeding 90%, confirming the models’ effectiveness in environments with low JNR. Zhang et al. [31] integrated target detection and recognition by adopting the YOLO network to extract information about jamming signals from STFT images, achieving both precision and recall rates above 85% when the JNR exceeds 0 dB. Recent studies have also explored few-shot and data-limited scenarios in radar jamming recognition. Approaches incorporating self-attention, multimodal fusion, and knowledge distillation have been proposed to enhance recognition under scarce training samples [32,33]. Transfer learning and pretraining strategies have further improved generalization, enabling robust performance across complex or low-JNR environments [34,35]. In addition, lightweight and multi-label recognition models have been developed to address overlapping jamming types and reduce model complexity [36,37]. Overall, these works highlight the growing interest in improving data efficiency and robustness in jamming recognition.

Despite recent progress in radar jamming recognition, several challenges remain unresolved, especially in the context of compound jamming under low-JNR conditions. Existing methods often rely on single-domain or weakly coupled features, which limit their ability to fully characterize the complex time–frequency structures introduced by compound suppressive and deceptive jamming. Moreover, many deep learning-based approaches achieve improved recognition performance at the cost of high model complexity, making them unsuitable for real-time or resource-constrained ECCM applications. To address the problem of recognizing compound jamming signals caused by the superposition of multiple jamming types, this paper proposes a lightweight compound jamming recognition network based on multi-feature fusion. The main contributions and innovations of this paper are as follows:

1. Based on various suppressive and deceptive jamming signals, a compound jamming signal dataset is constructed, and three complementary time–frequency transformations are used to systematically preprocess the dataset;
2. To improve the recognition accuracy of compound jamming signals, a multi-feature fusion network combined with an attention mechanism is proposed, which is capable of extracting both global and local features and performing adaptive weighting fusion across multiple time–frequency maps;
3. To reduce the overall computational complexity of the network while ensuring its recognition robustness in complex electromagnetic environments, an improved lightweight recognition network is designed.

The structure of this paper is as follows. Section 2 establishes the mathematical models of the radar transmitted signal, suppressive jamming, and deceptive jamming. Section 3 details the preprocessing methodology applied to the single-jamming dataset. Section 4 proposes a multi-feature fusion network and explains the function of each component. In Section 5, a lightweight recognition network model is introduced. Section 6 validates the proposed model through simulations, including the specification of parameter settings, comparative experiments, and analysis of results. Section 7 provides the conclusions and research contributions of this paper.

## 2. Mathematical Modeling of Radar Jamming Signals

Radar jamming can be classified into various categories, with one common distinction being active and passive jamming, depending on the source of the jamming energy. This paper focuses on active jamming, which is further divided into suppressive and deceptive jamming based on the interference mechanism. Suppressive jamming masks real target echoes with noise, reducing the signal-to-noise ratio (SNR) and hindering the radar’s ability to extract detailed target information. Typical forms include amplitude-modulation noise (AMN) and frequency-modulation noise (FMN) jamming. In contrast, deceptive jamming uses DRFM cognitive jammers to create false targets that are similar to real targets, causing confusion in the radar system. This category includes techniques such as interrupted sampling and repeating jamming (ISRJ), smeared spectrum (SMSP) jamming, range gate pull-off (RGPO), and velocity gate pull-off (VGPO).

**Figure 1:**
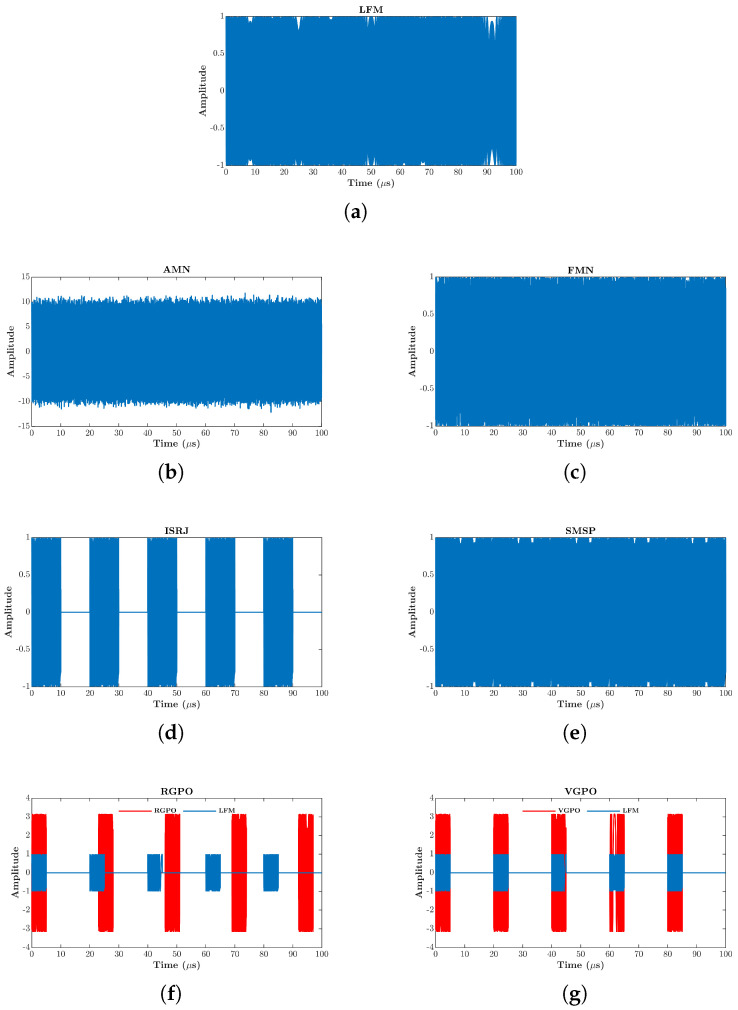
Time-domain waveform map of seven types of jamming signals. (**a**) LFM. (**b**) AMN. (**c**) FMN. (**d**) ISRJ. (**e**) SMSP. (**f**) RGPO. (**g**) VGPO.

This section establishes the mathematical models of the radar transmitted signal, suppressive jamming, and deceptive jamming, with their time-domain waveforms visualized in Figure 1.

### 2.1. Radar Transmitted Signal

The linear frequency-modulation (LFM) signal, valued for its high resolution and anti-jamming robustness in modern radar systems, is utilized as the transmission waveform in this paper. The time-domain expression of the LFM signal is given as

(1) $$s\left(t\right)=Arect\left(\frac{t}{T}\right)\exp\left[j2\pi \left(f_{0}t+\frac{1}{2}kt^{2}\right)\right]$$

where *A* denotes the amplitude, $f_{0}$ represents the carrier frequency, *t* is the time variable, and k=B/T is the frequency-modulation slope, where *B* and *T* are the signal bandwidth and pulse width, respectively. The mathematical formula for the rectangular function is

(2) $$rect\left(\frac{t}{T}\right)=\left\{\begin{array}{cc} 1,\; & -T/2\leq t\leq T/2\\ 0,\; & otherwise \end{array}\right.$$

Active jamming signals can be modeled and simulated using the radar transmission waveform as a basis.

### 2.2. Radar Suppressive Jamming

#### 2.2.1. Amplitude-Modulation Noise Jamming

AMN jamming is achieved by modulating a noise signal onto the carrier, which causes the signal’s amplitude to vary with the noise fluctuations. Its signal expression can be written as

(3) $$J\left(t\right)=\left[U_{0}+U_{n}\left(t\right)\right]cos\left[w_{j}t+\varphi \right]$$

where $U_{n}$ corresponds to a normal distribution with a mean of 0 and variance $\sigma^{2}$. $U_{0}$ and $w_{j}$ are the carrier voltage and carrier frequency, respectively, which are constants. The phase φ is uniformly distributed over the interval $\left[0,2\pi \right]$ and is independent of $U_{n}\left(t\right)$. With simple hardware implementation, AMN is widely used in electronic countermeasures.

#### 2.2.2. Frequency-Modulation Noise Jamming

FMN jamming is a type of jamming in which a noise signal modulates the carrier by varying its frequency while maintaining a fixed amplitude. Its mathematical expression is

(4) $$J\left(t\right)=U_{0}\cos\left[w_{j}t+2\pi K_{\mathrm{FM}}\int_{0}^{t}u\left(t^{\prime }\right)\;dt^{\prime }+\varphi \right]$$

where u(t) conforms to a normal distribution. $U_{0}$ and $w_{j}$ are the carrier voltage and carrier frequency, respectively, which are constants. $K_{\mathrm{FM}}$ is the frequency-modulation slope. The phase φ is uniformly distributed over the interval $\left[0,2\pi \right]$ and is independent of $U_{n}\left(t\right)$. The wide frequency range enables it to effectively mask the target signal.

### 2.3. Radar Deceptive Jamming

#### 2.3.1. Interrupted Sampling Repeater Jamming

ISRJ is generated using a DRFM jammer to sample and retransmit the transmitted signal multiple times within a pulse repetition period. The time-domain model for ISRJ is expressed as

(5) $$\begin{array}{cc} J_{\mathrm{ISRJ}}\left(t\right) & =\sum_{n=1}^{N}\frac{1}{\sqrt{\tau }}rect\left(\frac{t-\frac{\tau }{2}-\left(n-1\right)T_{s}}{T}\right)\\ \; & \;\times \;e^{j2\pi \left(f_{0}t+\frac{K}{2}t^{2}\right)} \end{array}$$

where *N* represents the number of jamming samples. τ is the width of the interrupted sampled pulse. $T_{s}$ and *T* denote the pulse width of the transmitted signal and the sampling period, respectively.

#### 2.3.2. Smeared Spectrum Jamming

SMSP jamming signals consist of *n* identical sub-pulses, each with a pulse width of 1/n of the transmitted signal, while the modulation slope is n times that of the transmitted signal. The mathematical expression for SMSP jamming is

(6) $$\begin{array}{cc} J\left(t\right) & =\exp\left(j2\pi f_{0}t+j\pi k^{\prime }t^{2}\right),k^{\prime }=nk,0\leq t\leq \frac{T}{n}\\ \; & J_{\mathrm{SMSP}}\left(t\right)=\sum_{i=1}^{n}J\left(t-\left(i-1\right)\frac{T}{n}\right) \end{array}$$

where *T* and *K* denote the pulse width and frequency-modulation slope of the transmitted signal, respectively.

#### 2.3.3. Gate Pull-Off Jamming

Gate pull-off jamming is a common deceptive jamming technique designed to resist radar tracking systems. Based on different tracking mechanisms, it can be classified into RGPO and VGPO [38,39]. RGPO jamming works by generating false echo signals to mislead the radar into acquiring incorrect range information, ultimately disrupting its ability to accurately track the target. Similarly, VGPO jamming exploits Doppler frequency spoofing to distort velocity information, thereby inducing equivalent disruption to radar systems. They are both periodic processes, consisting of three phases: halting of the pull-off, pulling off, and closing:

(7) $$\begin{array}{cc} J\left(t\right)\; & =A\times \exp\left[j\left(\varphi \left(t-t_{0}-\Delta t-\Delta t_{J}\left(t\right)\right)+\varphi \right)\right]\\ \; & \;\times \exp\left[j2\pi \Delta f_{J}\left(t\right)t\right] \end{array}$$

where *A* is the amplitude. $\varphi \left(t-t_{0}-\Delta t-\Delta t_{J}\left(t\right)\right)$ serves as the phase term that captures phase fluctuations. $t_{0}$ denotes the moment the radar pulse is received. The parameter Δt represents the inherent time delay introduced by the jammer, while $\Delta t_{J}$ accounts for additional timing variations, such as range-induced lag or other delay-related effects. φ corresponds to the initial phase of the jamming signal. Furthermore, $\Delta f_{J}\left(t\right)$ characterizes the frequency-shift component, which reflects the Doppler effect associated with the jamming signal.

## 3. Signal Preprocessing

This section methodically examines the coupling mechanisms of compound jamming signals and analyzes three time–frequency analysis methods used for jamming recognition.

### 3.1. Radar Compound Jamming

Compound jamming can be classified into three primary forms based on their generation mechanisms: additive, multiplicative, and convolutional. Among these, multiplicative and convolutional approaches involve high computational complexity and impose stringent demands on the real-time processing capabilities of jamming equipment. Additive compound jamming is the most common form in modern electronic warfare and consists of a linear superposition of suppressive jamming and deceptive jamming in the time domain. This type of jamming significantly degrades the target detection performance of radar receivers. Given its high prevalence in operational scenarios, this study focuses on an in-depth analysis of this jamming category, whose mathematical representation is given by

(8) $$J\left(t\right)=J_{1}\left(t\right)+J_{2}\left(t\right)$$

where $J_{1}\left(t\right)$ and $J_{2}\left(t\right)$ represent suppressive and deception jamming, respectively. This paper focuses on the recognition of additive compound jamming signals. Specifically, the compound categories include AMN + ISRJ, FMN + ISRJ, ISRJ + RGPO, ISRJ + SMSP, ISRJ + VGPO, SMSP + RGPO, and SMSP + VGPO, which are designated as classes 1 through 7, respectively.

### 3.2. Signal Transformation

Among the various time–frequency analysis techniques, this study focuses on three representative methods for feature extraction.

#### 3.2.1. Short-Time Fourier Transform

The short-time Fourier transform (STFT) is a classical and computationally efficient time–frequency analysis technique that applies the Fourier transform within a fixed-length sliding window [10]. Its mathematical formula is

(9) $$STFT\left(t,f\right)=\int_{-\infty }^{+\infty }x\left(\tau \right)h\left(\tau -t\right)e^{-j2\pi f\tau }d\tau$$

where $x\left(t\right)$ is a signal in the time domain and $h\left(t\right)$ is a window function.

#### 3.2.2. Smoothed Pseudo-Wigner–Ville Distribution

Smoothed Pseudo-Wigner–Ville Distribution (SPWVD) is derived from the Wigner–Ville distribution and improves time–frequency representation by suppressing cross-term interference through smoothing kernels [12]. Compared with STFT, SPWVD exhibits superior time–frequency resolution and better energy concentration, which is beneficial for characterizing rapidly varying components in compound jamming. However, SPWVD incurs higher computational complexity. SPWVD is defined as

(10) $$\begin{array}{cc} SPWVD\left(t,f\right) & =\overset{+\infty }{\underset{-\infty }{\;\int \int \;}}x\left(t+\frac{\tau }{2}+\theta \right)x^{*}\left(t-\frac{\tau }{2}+\theta \right)\\ \; & \;\times \;h\left(\theta \right)g\left(\tau \right)e^{-j2\pi f\tau }d\tau d\theta \end{array}$$

where $h\left(\theta \right)$ and $g\left(\tau \right)$ are window functions.

#### 3.2.3. Continuous Wavelet Transform

The wavelet transform is another commonly used method for time–frequency analysis. It can be divided into the discrete wavelet transform (DWT) and the continuous wavelet transform (CWT). A key feature of CWT is that it is multi-scale, providing high time resolution at higher frequencies and low frequency resolution at lower frequencies. This makes it especially effective for analyzing non-stationary signals, with applications in weak target recognition [15]. The expression of CWT is given by

(11) $$W\left(s,p\right)=\frac{1}{\sqrt{s}}\int_{-\infty }^{+\infty }x\left(t\right)\psi^{*}\left(\frac{t-p}{s}\right)dt$$

where $x\left(t\right)$ is a radar signal. *s* and *p* denote the scale and translation factors, respectively, and $\psi^{*}$ is the complex conjugate of the mother wavelet.

In summary, STFT, SPWVD, and CWT provide complementary time–frequency representations in terms of computational efficiency, resolution, and multi-scale analysis capability. Consequently, these three time–frequency transformation methods are selected to construct a complementary time–frequency feature analysis framework, providing a solid foundation for subsequent feature fusion and jamming recognition tasks.

## 4. Design of Feature Fusion Network

**Figure 2:**
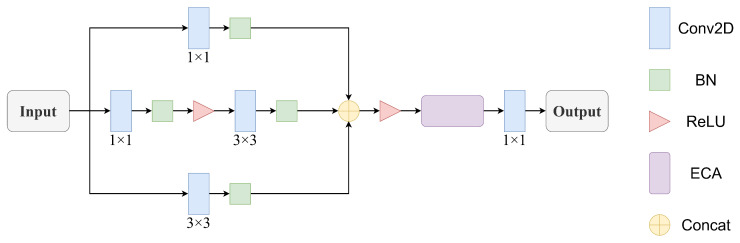
MSE feature fusion network.

Based on the three time–frequency representations obtained in the preprocessing stage, this section proposes a multi-branch parallel feature fusion network, named MSE, which architecture is illustrated in Figure 2, to perform multi-scale feature extraction for each type of time–frequency image. In addition, an attention mechanism is incorporated to enhance the model’s sensitivity and representational capability with respect to critical jamming features. In particular, the first branch uses a 1 × 1 two-dimensional convolutional (Conv2d) layer to reduce channel dimensionality and capture global feature correlations with low computational cost. The second branch employs a 3 × 3 Conv2d layer to expand the receptive field and extract local spatial features from jamming time–frequency maps. The third branch cascades 1 × 1 and 3 × 3 Conv2d layers to balance dimensionality reduction and spatial feature extraction, with a ReLU activation function inserted to introduce nonlinearity. After concatenating the outputs of the three branches, an Efficient Channel Attention (ECA) [40] module is embedded. It employs a one-dimensional convolution with an adaptive kernel size to capture local cross-channel dependencies. Specifically, global average pooling is first applied to the concatenated feature maps to compress spatial information into channel-wise statistics. Subsequently, a one-dimensional convolution is used to model correlations among neighboring channels, followed by a sigmoid function to generate channel attention weights. These weights are then multiplied with the original concatenated features, enabling the network to adaptively highlight effective channels that contribute to compound jamming recognition while suppressing redundant channels. This design provides more discriminative feature representations for subsequent compound jamming recognition, ultimately strengthening intelligent countermeasure capability in complex electromagnetic environments.

Taking the STFT channel as an example, let the input feature map be $X=R^{C\times H\times W}$, where *C*, *H*, and *W* represent the number of channels, the height, and the width, respectively.

The initial branch transformation consists of a linear 1×1 convolution followed by batch normalization, formally expressed as

(12) $$F_{1}=BN^{\left(1\right)}\left(W_{1\times 1}^{\left(1\right)}*X+b_{1}\right)$$

where $W_{1\times 1}^{\left(1\right)}$ denotes the trainable weight matrix in the convolutional layer and ∗ denotes the convolution operation. $b_{1}$ is the bias term. BN (batch normalization) is a normalization technique designed to accelerate neural network training and improve model stability. The superscript indicates the branch index, and the parameter notation remains consistent in subsequent derivations without repeated definitions.

Based on the structure of the first branch, the second branch employs 3×3 convolutions instead of 1×1 kernels to expand the receptive field to capture richer spatial context information:

(13) $$F_{2}=BN^{\left(2\right)}\left(W_{3\times 3}^{\left(2\right)}*X+b_{2}\right)$$

The third branch combines the strengths of the preceding branches through a sequential cascade of 1×1 and 3×3 convolutional kernels. This architecture not only enables efficient channel dimension modulation but also extracts local spatial features, achieving an optimal balance between computational efficiency and feature representation. The operations can be mathematically represented sequentially as follows:

(14) $$\begin{array}{cc} F_{3}^{\prime }\; & =\sigma_{1}\left(BN_{1}^{\left(3\right)}\left(W_{1\times 1}^{\left(3\right)}*X+b_{3}\right)\right)\\ \; & F_{3}=BN_{2}^{\left(3\right)}\left(W_{3\times 3}^{\left(3\right)}*F_{3}^{\prime }+b_{4}\right) \end{array}$$

where $\sigma_{1}\left(•\right)$ is a nonlinear activation layer implemented as a ReLU function.

The feature maps produced by the three parallel branches are concatenated along the channel dimension and subsequently processed by a ReLU activation layer:

(15) $$F=\sigma_{2}\left(Concat\left(F_{1},F_{2},F_{3}\right)\right)$$

where $\sigma_{2}\left(•\right)$ is another nonlinear activation layer.

The fused features are then passed into the ECA module to improve inter-channel dependencies. The ECA module produces attention weights $W=R^{C}$ via

(16) $$W=\mathrm{Sigmoid}\left(\mathrm{Conv}{\mathrm{lD}}_{k}\left(\mathrm{GAP}(F)\right)\right)$$

In the activation function, Sigmoid(•) denotes the sigmoid function, GAP(•) denotes global average pooling, and $\mathrm{Conv}{\mathrm{lD}}_{k}\left(•\right)$ implements a 1D convolution with kernel size k.

The refined features are obtained as

(17) $$F_{\mathrm{ECA}}=W⊗F$$

where ⊗ indicates channel-wise multiplication. The ECA module achieves lower computational complexity while maintaining performance. By integrating the fusion network, it improves channel attention discrimination and improves representational capacity. The output of this channel is generated by applying channel-wise dimensionality reduction via a 1×1 convolution to the fused features:

(18) $$Y_{\mathrm{STFT}}=W_{1\times 1}^{\left(4\right)}*F_{\mathrm{ECA}}+b_{4}$$

**Figure 3:**
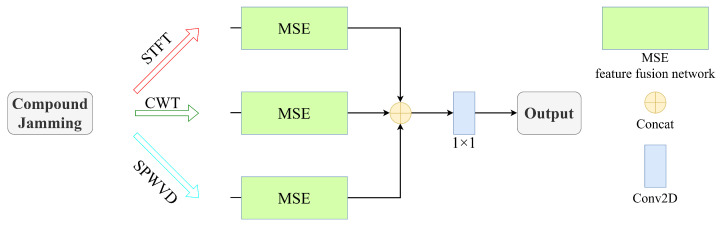
Overall structure of the network.

Similarly, the output features of the other two channels are obtained as $Y_{\mathrm{CWT}}$ and $Y_{\mathrm{SPWVD}}$, respectively. To enhance the integration of multi-domain time–frequency information, weighted concatenation with distinct coefficients is applied to the three-channel features, resulting in a multi-scale fused feature map. This operation is illustrated in Figure 3 and can be formulated as

(19) $$Y^{\prime }=\alpha Y_{\mathrm{STFT}}+\beta Y_{\mathrm{CWT}}+\gamma Y_{\mathrm{SPWVD}}$$

where α, β, and γ are optimal weighting coefficients learned during training.

Ultimately, $Y^{\prime }$ undergoes dimensionality reduction to generate the fused image Y, which serves as the output of the fusion module and simultaneously as the input to the recognition network.

## 5. Design of Lightweight Recognition Network

**Figure 4:**
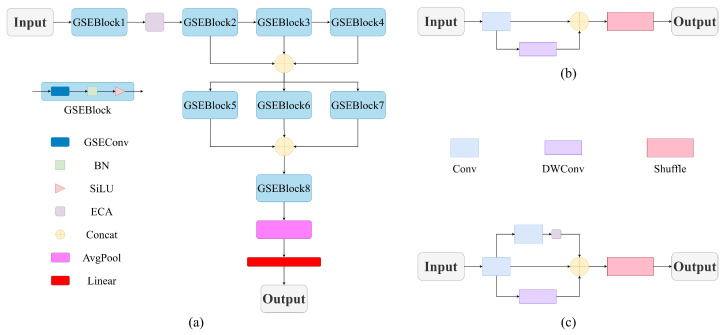
The proposed lightweight recognition network, GSENet. (**a**) Overall framework. (**b**) Original GSConv. (**c**) Improved GSEConv.

The images generated by the feature fusion network described above are employed for compound jamming recognition. A lightweight recognition network, GSENet, is proposed based on the GSEConv modules, which are optimized variants of the original GSConv [41], as illustrated in Figure 4a. The overall GSENet network follows a hierarchical feature extraction strategy and consists of four main components: a series of GSEBlock modules stacked in both cascaded and parallel configurations, an ECA module, an average pooling (AvgPool) layer, and a fully connected (FC) layer.

Specifically, each GSEBlock is constructed using the proposed GSEConv for feature transformation, followed by a BN layer to improve training stability and a SiLU activation function to enhance nonlinear representation. Compared with ReLU, SiLU provides smoother gradients and improved feature expressiveness, which contributes to higher recognition accuracy. The first GSEBlock module performs initial feature extraction, followed by the ECA module, which reinforces feature correlations by modeling interdependencies between channels. Subsequently, three cascaded GSEBlock modules progressively extract features from low to high levels, and their outputs are concatenated to aggregate multi-scale spatial information. Then, the other three parallel GSEBlock modules process the concatenated features to capture richer semantic information, and their outputs are concatenated again for deeper fusion. Finally, a GSEBlock module integrates and refines the fused semantic features.

The original GSConv module structure is presented in Figure 4b. It first performs feature extraction on the input image through standard convolution for downsampling, followed by depth-wise convolution (DWConv) processing. The outputs from the two convolutional branches are organized along the channel dimension and restructured through channel shuffling to enhance feature integration.

However, the original GSConv still suffers from some information loss. To mitigate this issue, the GSEConv module was proposed, as illustrated in Figure 4c. In addition to the original two branches, a third branch incorporating standard convolution, followed by an ECA module, is introduced to further enhance multi-scale spatial representation and strengthen channel-wise feature interdependencies.

The features extracted by multiple GSEBlocks are progressively processed and aggregated across network layers. This combination of multi-level features allows the network to capture both low-level and high-level information, enabling better representational capability and discrimination of learned representations.

After feature aggregation, an average pooling operation is applied to reduce spatial dimensions while retaining salient information. Finally, a fully connected linear layer maps the aggregated global feature vectors to the target label space for recognition prediction.

## 6. Simulation Experiments

### 6.1. Dataset Construction

The jamming waveforms specified in Section 2 and their compound combinations were simulated in MATLAB R2022b (MathWorks, Natick, MA, USA), with the complete parameter settings listed in Table 1.

For the first jamming signal, the carrier frequency and bandwidth were varied from 6 to 15 MHz in increments of 3 MHz. The second jamming signal employed a carrier frequency sweep from 4 to 16 MHz in 3 MHz increments and a bandwidth sweep from 5 to 15 MHz in 5 MHz increments. The parameter N, representing either the sampling count of ISRJ or the sub-pulse number of SMSP, was varied from 4 to 10 in increments of 2 and from 3 to 6 in increments of 1, respectively. In addition, the system maintained a fixed sampling rate and pulse width of 100 MHz and 5 us. The total duration of the jamming was 100 us.

In the experiment, the JNR and AWGN were configured as background noise, swept from −10 dB to 10 dB in 4 dB increments. The mathematical formulation is given by

(20) $$JNR=10\log_{10}\frac{P_{J}}{P_{AWGN}}$$

where $P_{J}$ is the power of the compound jamming and $P_{AWGN}$ represents the AWGN power.

### 6.2. Simulation Environment and Parameter Settings

All network designs and experiments were conducted on a workstation running Ubuntu 20.04 (Canonical Ltd., London, UK). The deep learning models were implemented using Python 3.8 (Python Software Foundation, Wilmington, DE, USA) and the PyTorch 2.0.0 framework (Meta Platforms Inc., Menlo Park, CA, USA). The hardware platform consisted of a 16-core Intel Xeon Gold 6430 CPU (Intel Corporation, Santa Clara, CA, USA) and an NVIDIA RTX 4090 GPU with 24 GB memory (NVIDIA Corporation, Santa Clara, CA, USA), supporting CUDA 11.8.

By jointly considering model complexity, training stability, and recognition accuracy, an appropriate training strategy was adopted in this paper. The fused dataset was divided into training, validation, and testing sets with a ratio of 6:2:2. The recognition network was trained for 100 epochs with a batch size of 8, and cross-entropy loss was used as the objective function. The Adam optimizer (learning rate of 0.001) was selected for its adaptive learning rate capability, which facilitates stable and efficient convergence during training. The performance of the network was assessed on test data using recognition accuracy, determined as the ratio of accurate predictions to total test samples.

### 6.3. Experimental Results and Analysis

#### 6.3.1. Comparisons with Machine Learning Methods

**Figure 5:**
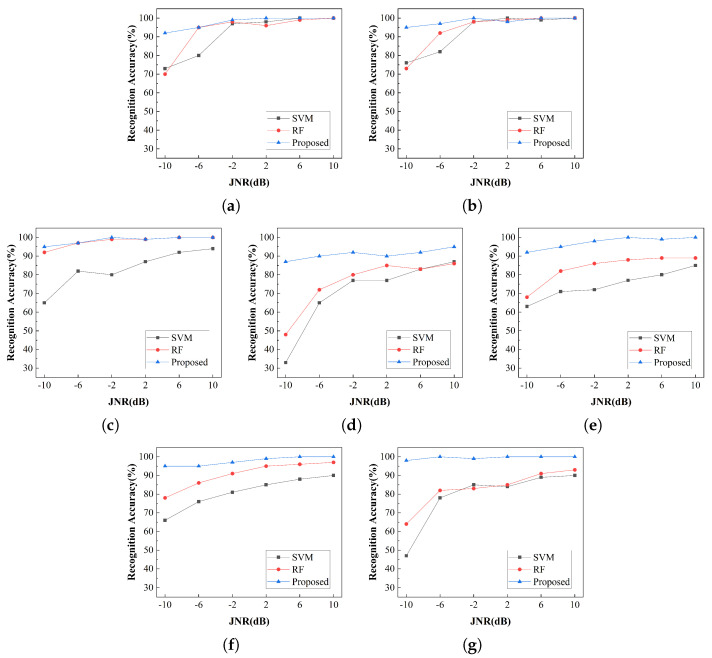
Recognition accuracy for different compound jamming signal types. (**a**) AMN + ISRJ. (**b**) FMN + ISRJ. (**c**) ISRJ + RGPO. (**d**) ISRJ + SMSP. (**e**) ISRJ + VGPO. (**f**) SMSP + RGPO. (**g**) SMSP + VGPO.

A comparative analysis was conducted between the proposed method and two traditional machine learning models (SVM and RF) to recognize seven types of compound jamming signals under different JNR conditions. The recognition accuracy performance is presented in Figure 5, where Figure 5a–g correspond to the seven compound jamming signals in sequential order. Experimental results show that the recognition accuracy for all seven categories of compound jamming improved as the JNR increased. Across most JNR levels, the proposed method achieved the highest accuracy, followed by RF, while SVM exhibited relatively lower performance. These findings demonstrate that the proposed method exhibits superior robustness and feature-extraction capability in the presence of compound jamming signals.

In scenarios involving combined suppressive and deceptive jamming signals, such as AMN + ISRJ and FMN + ISRJ, both SVM and RF techniques exhibited low recognition accuracy at a JNR of -10dB, whereas the proposed method achieved significantly higher performance. This indicates that traditional approaches struggle with feature extraction under strong noise conditions, whereas the proposed method shows superior adaptability to such complex jamming environments. For compound jamming signals formed by superimposed deceptive jamming types, the proposed method demonstrates significant advantages. It can achieve a perfect recognition accuracy of 100% even under relatively low-JNR conditions, confirming its ability to effectively distinguish different types of deceptive jamming and providing a reliable and efficient solution for anti-jamming countermeasures.

#### 6.3.2. Comparisons with Deep Learning Networks

In addition to traditional machine learning recognition methods, three deep learning based recognition networks were compared with the proposed GSENet: the standard CNN architecture [21], ResNet50 [42], and the network using the original GSConv module [41]. The accuracy results are reported in Table 2. Experimental results show that the performance gains of the proposed GSENet were not uniformly distributed across all compound jamming classes but were particularly pronounced for classes 4 and 5, corresponding to more complex deceptive jamming combinations. These classes exhibit stronger non-stationary and overlapping time–frequency features, making them more challenging for conventional networks to discriminate. The improved accuracy in these cases demonstrates that the proposed GSEConv module enhances the network’s ability to capture different features in complex time–frequency structures.

Although ResNet50 achieved competitive recognition accuracy, its extremely high parameter count makes it less suitable for ECCM applications. The standard CNN also performed relatively well; however, it still required several times more parameters than GSENet. Compared with the network based on the original GSConv module, GSENet achieved consistent accuracy improvements across all seven classes with only a marginal increase in parameters. This indicates that the additional ECA-enhanced branch in GSEConv effectively compensates for information loss, improving feature-extraction capability without significantly increasing computational burden. Overall, the comparative results confirm that the proposed GSENet achieves higher recognition accuracy than other deep learning-based networks.

#### 6.3.3. Comparisons with Lightweight Networks

To further evaluate the effectiveness of the proposed lightweight recognition network, GSENet, under low-JNR conditions, comparative experiments were conducted with two representative lightweight networks: MobileNetV2 [43] and EfficientNet [44]. All networks were independently trained and tested on the seven types of compound jamming signals under three low-JNR scenarios: −10 dB, −6 dB, and −2 dB, following the same training strategy. The experimental results are shown in Table 3.

As presented in Table 3, the proposed GSENet achieved superior recognition accuracy across all seven compound jamming classes under the three low-JNR conditions. In particular, under the most challenging scenario of −10 dB, GSENet exhibited a clear performance advantage for classes 3 to 5, where MobileNetV2 and EfficientNet suffered from noticeable accuracy degradation. It is also worth noting that the proposed network achieved these performance gains with a significantly smaller number of parameters, demonstrating a favorable trade-off between recognition accuracy and model complexity. These results validate the effectiveness of the proposed lightweight recognition network, GSENet, for compound jamming recognition under low-JNR conditions.

## 7. Conclusions

In this paper, a lightweight radar compound jamming recognition network based on multi-feature fusion was proposed to address the challenges of insufficient feature representation and high model complexity under low-JNR conditions. First, a dataset of additive compound jamming signals comprising seven combinations of suppressive and deceptive jamming was constructed, and three complementary time–frequency transformation methods, namely STFT, SPWVD, and CWT, were employed for preprocessing. Based on these preprocessed time–frequency maps, a multi-branch parallel feature fusion network, MSE, integrated with an ECA mechanism was designed. MSE extracts multi-scale features in parallel, and the ECA module adaptively enhances discriminative channel information while suppressing redundancy. A weighted fusion strategy further integrates features from different time–frequency domains, providing more informative representations for subsequent recognition tasks. To balance recognition accuracy and model complexity, an improved lightweight recognition network named GSENet was proposed based on the optimized GSEConv module. By introducing a third branch with ECA, GSEConv compensates for information loss of the original GSConv, while the combination of cascaded and parallel GSEBlocks enables hierarchical multi-scale feature aggregation. Experimental results show that GSENet achieves an average recognition accuracy of over 87% for seven compound jamming types, outperforming traditional machine learning methods such as SVM and RF, as well as mainstream deep learning models, including the standard CNN and ResNet50. Compared with representative lightweight networks such as MobileNetV2 and EfficientNet, GSENet maintains higher accuracy while reducing the parameter count to below 0.14 M, achieving a better balance between recognition accuracy and model complexity. These significant advantages indicate that GSENet is well suited for deployment on resource-constrained radar platforms, enabling efficient jamming recognition and improving the survivability of radar systems in complex electromagnetic environments, thereby providing meaningful support for practical ECCM applications.

## Figures and Tables

**Table 1:** Simulation parameters for compound jamming.

Type	Parameters (MHz)	Value Ranges
$J_{1}\left(t\right)$	Carrier Frequency	6 to 15
	Bandwidth	6 to 15
$J_{2}\left(t\right)$	Carrier Frequency	4 to 16
	Bandwidth	5 to 15

**Table 2:** Recognition accuracy results of various neural network models.

	Class 1	Class 2	Class 3	Class 4	Class 5	Class 6	Class 7	Params (M)
CNN [21]	[0.92,0.97,1,1,1,1]	[0.96,0.99,1,1,0.99,1]	[0.97,0.99,1,1,0.99,1]	[0.83,0.88,0.93,0.95,0.92,0.95]	[0.93,0.95,0.99,1,1,1]	[0.95,0.97,1,1,0.98,0.99]	[1,0.98,0.99,1,1,1]	0.72
ResNet50 [42]	[0.91,0.96,0.99,1,1,1]	[0.93,0.95,1,1, 0.98,1]	[0.95,0.98,1,1,1,1]	[0.8,0.87,0.9,0.9,0.9,0.93]	[0.9,0.94,0.96,1,1,0.97]	[0.93,0.96,0.99,0.99,1,1]	[0.95,0.99,1,1,1,1]	25.56
GSConv [41]	[0.90,0.93,0.95,0.95,1,0.98]	[0.89,0.9,0.88,0.93,0.99,1]	[0.9,0.93,0.95,0.95,0.96,0.94]	[0.8,0.81,0.9,0.9,0.92,0.92]	[0.9,0.92,0.95,0.98,1,1]	[0.91,0.93,0.94,0.97,1,1]	[0.97,0.99,1,1,0.97,1]	0.05
**Proposed**	**[0.92,0.95,0.99,1,1,1]**	**[0.95,0.97,1,0.98,1,1]**	**[0.95,0.97,1,0.99,1,1]**	**[0.87,0.9,0.92,0.9,0.92,0.95]**	**[0.92,0.95,0.98,1,0.99,1]**	**[0.95,0.95,0.97,0.99,1,1]**	**[0.98,1,0.99,1,1,1]**	**0.14**

**Table 3:** Recognition accuracy results of lightweight networks under low-JNR conditions.

	Class 1	Class 2	Class 3	Class 4	Class 5	Class 6	Class 7	Params (M)
MobileNetV2 [43]	[0.89,0.95,0.97]	[0.93,0.93,0.95]	[0.91,0.95,1]	[0.68,0.78,0.85]	[0.88,0.91,0.98]	[0.91,0.96,0.99]	[0.96,0.98,0.98]	0.32
EfficientNet [44]	[0.93,0.95,0.98]	[0.89,0.92,0.97]	[0.90,0.91,0.95]	[0.74,0.82,0.90]	[0.88,0.92,0.94]	[0.91,0.93,0.97]	[0.96,0.99,1]	4.02
**Proposed**	**[0.92,0.95,0.99]**	**[0.95,0.97,1]**	**[0.95,0.97,1]**	**[0.87,0.9,0.92]**	**[0.92,0.95,0.98]**	**[0.95,0.95,0.97]**	**[0.98,1,0.99]**	**0.14**

## Data Availability

The data are not publicly available due to research restrictions.
